# The scent gland chemistry of neogoveid cyphophthalmids (Opiliones): an unusual methyljuglone from *Metasiro savannahensis*

**DOI:** 10.1007/s00049-019-00288-y

**Published:** 2019-09-26

**Authors:** Günther Raspotnig, Felix Anderl, Ronald M. Clouse

**Affiliations:** 1 Institute of Biology, University of Graz, Universitätsplatz 2, 8010 Graz, Austria; 2 Department of Bioinformatics and Genomics, University of North Carolina at Charlotte, University City Blvd., 9201, Charlotte, NC 28223 USA; 3 Present Address: American Museum of Natural History, 79th St. at Central Park West, New York, NY 10024 USA

**Keywords:** Cyphophthalmi, Chemosystematics, Hydroxymethylnaphthoquinone, Scent gland secretion, Antibiotic

## Abstract

While the chemistries of scent gland secretions from a few selected species of three families of Cyphophthalmi, namely Sironidae, Pettalidae, and Stylocellidae, have already been reported and found to consist of complex blends of naphthoquinones and methyl ketones, nothing is known about the other families. We here report on the secretions of *Metasiro savannahensis* Clouse and Wheeler (Zootaxa 3814:177–201, 2014), a first representative of the family Neogoveidae. The secretions from males, females and one juvenile were extracted and analyzed by gas chromatography–mass spectrometry. Twenty-five compounds were identified, all of which belong to the chemical classes of naphthoquinones and methyl ketones, confirming a hypothesized chemical uniformity of cyphophthalmid exudates. One major naphthoquinone compound, however, was new for cyphophthalmids and for arthropod exocrine secretions in general: a methyljuglone isomer, 6-methyljuglone (= 6-MJ; iupac name: 5-hydroxy-6-methyl-1,4-naphthoquinone), amounted for about 20% of the secretion and was eventually identified by synthesis. Hydroxy-naphthoquinones and their derivatives are known to possess a variety of antibiotic effects, probably enhancing the antimicrobial/antifungal potential of the *Metasiro*-secretion. Currently, without further data on neogoveids, the compound represents a chemical autapomorphy of *M. savannahensis*, and—just as the strange chloro-naphthoquinones of Sironidae and Pettalidae—adds to the repertoire of unusual naphthoquinone compounds across the Cyphophthalmi.

**Electronic supplementary material:**

The online version of this article (10.1007/s00049-019-00288-y) contains supplementary material, which is available to authorized users.

## Introduction

An important synapomorphic character of all harvestmen is the presence of so-called scent glands, which constitute the largest exocrine system of this arachnid order. Scent gland secretions are considered to be mainly for defense (e.g., Martens 1978; Holmberg 1986; Eisner et al. 2004: Machado et al. 2005), and their chemistry has been investigated since the 1950s, revealing a large array of unusual natural compounds, including rare quinones, ketones, and nitrogen-containing compounds (Gnaspini and Hara 2007; Raspotnig 2012). Most interestingly, compound profiles from scent glands are taxon specific and have emerged as a tool to trace the evolutionary history of secretion chemistry across Opiliones (e.g., Raspotnig et al. 2015, 2017).

The smallest suborder of Opiliones, comprising only about 200 named species (Kury 2013), is the leaf litter- and cave-dwelling Cyphophthalmi. These short-legged, mite-like opilionids possess the most conspicuous scent gland system of all harvestmen: scent gland openings are located on tubercles, so-called ozophores, and secretions are administered to offenders by “leg dabbing”—a behavior which involves the transfer of secretion droplets from the top of ozophores to body parts of the aggressor by legs, mainly by the tip of leg II (Juberthie 1961).

The chemistry of two cyphophthalmid species of family Sironidae was initially investigated in 2005 and was found to be surprisingly complex, comprising more than 20 compounds, all of which belonged to the chemical classes of naphthoquinones and methyl ketones (Raspotnig et al. 2005). Among naphthoquinones, unusual chloro-naphthoquinones, at this time unique in the animal kingdom, were reported. Since then, only two further investigations, one on the chemistry of Stylocellidae (Jones et al. 2009) and a second one on the chemistry of Pettallidae (Raspotnig et al. 2012) have been conducted, both studies confirming the basic naphthoquinone/methyl ketone-organization of cyphophthalmid secretions, though in species-specific patterns. In total, the secretions of four species from three families, Sironidae, Stylocellidae, and Pettallidae, have been analyzed. According to current views on the internal systematics of Cyphophthalmi, three further families are recognized: Troglosironidae (restricted to New Caledonia), Ogoveidae (Africa), and Neogoveidae (South America, eastern North America and West Africa) (e.g., Giribet et al. 2012; Fernandez et al. 2017). The scent gland chemistry of these three families is still enigmatic.

We here report on the scent glands of a first representative of Neogoveidae, *Metasiro savannahensis*. *Metasiro* is the northern most neogoveid genus and is distributed in the southeastern United States.

## Materials and methods

A leaf-litter sample containing five males, ten females and one juvenile of *Metasiro savannahensis* Clouse and Wheeler, 2014 was collected in Jasper County, South Carolina USA, Kingfisher Pond, Savannah National Wildlife Refuge (32.18923; − 81.08008) in January 2015 by R. Clouse and M. Galac. Specimens were extracted from the leaf litter by hand, and scent gland secretions were collected either by whole body extractions of single individuals or by soaking the secretion on filter paper pieces directly after its discharge from ozopores (in both cases, 100 µl methylene chloride was used; extraction time: 15 min). Aliquots of extracts (1.5 µl) were used for gas chromatographic–mass spectrometric analyses (GC–MS) (see Raspotnig et al. 2017 for details on GC–MS instruments and conditions). Retention indices (RIs) for an apolar GC-phase (a ZB-5MS column from Phenomenex, Germany) were calculated according to Van den Dool and Kratz (1963), using an alkane standard (C_9_–C_36_). A comparison of gaschromatographic secretion profiles by Bray–Curtis dissimilarity (non-metric multidimensional scaling [nMDS] and PERMANOVA) was performed in PAST 3.25.

As references to identify compounds, we used the already fully analyzed secretion of *Cyphophthalmus duricorius* Joseph 1868 (Raspotnig et al. 2005). Authentic 7-methyljuglone (= 7-MJ; syn. 5-hydroxy-7-methyl-1,4-naphthoquinone) was extracted from the leaves of different sundew cultivars *(Drosera capensis* and a *D. aliciae* cultivar), bought in a market garden in Graz, Austria. 6-Methyljuglone (= 6-MJ; syn. 5-hydroxy-6-methyl-1,4-naphthoquinone) was synthesized according to a two-step procedure described by Mahapatra et al. (2007). Briefly, Friedel–Crafts type acylation of 4-chloro-2-methyl phenol with maleic anhydride in a molten eutectic mixture of aluminium chloride and sodium chloride yielded 8-chloro-5-hydroxy-6-methyl-1,4-naphthoquinone. Reductive de-chlorination mediated by tin(II) chloride and subsequent oxidation by iron(III) chloride provided the desired compound. Plumbagin (from *Plumbago indica*) (= 2-methyljuglone; syn. 5-hydroxy-2-methyl-1,4-naphthoquinone) as well as all reagents for 6-MJ-synthesis and a reference standard for 2-methoxy-1,4-naphthoquinone were purchased from Sigma (Vienna, Austria). NMR spectra for 7-MJ, 6-MJ, and plumbagin (1H, HSQC, HMBC) were recorded at the Institute of Pharmaceutical Sciences, University of Graz, Austria, using a 700 MHz Bruker Avance III spectrometer (supplement 1), and were found to be consistent with data from the literature (e.g., Mahapatra et al. 2007; Raj et al. 2011). Scanning electron micrographs (SEMs) were prepared at the Institute of Biology, Division of Plant Sciences, University of Graz, Austria, using a Philips XL30 ESEM at high vacuum mode and 20 kV accelerating voltage.

## Results and discussion

### Secretion chemistry and identification of 6-methyljuglone (6-MJ)

**Fig. 1:**
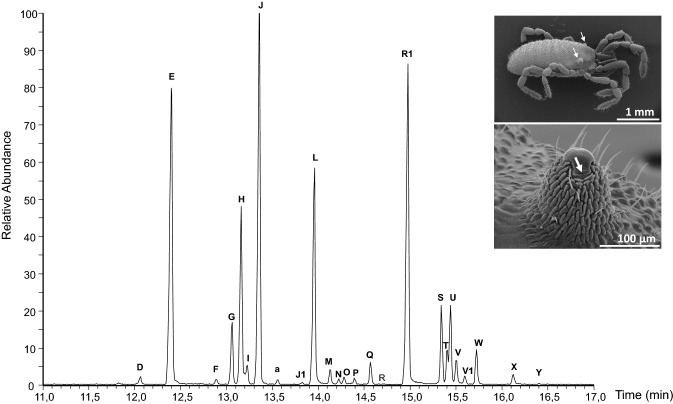
Characteristic gas chromatographic profile of the scent gland secretion of a specimen of *Metasiro savannahensis* (whole body extract of a male). The designation of peaks (compounds) by letters is in accordance with other publications on cyphophthalmid secretion chemistry (see Table 2). The insert (a female specimen) shows the position of ozophores (arrows), which protrude laterally from the prosoma (“type 2 ozophores”), as well details of the right ozophore, including the slit-like, anterolaterally directed opening (arrow)

Twenty-five components of extracts were assigned to the scent gland secretion of *Metasiro savannahensis* (Fig. 1). All compounds were identified by comparison to authentic standards or by comparison to the already known secretion of *Cyphophthalmus duricorius* (see Table 1). Naphthoquinones (5 compounds) as well as methyl ketones in a range from C_12_ to C_15_ (20 compounds) were present, making the extracts very similar to those of other cyphophthalmids (Table 2). One main constituent of the extracts (about 20% relative abundance), compound R1, was new for cyphophthalmid secretions. Compound R1 showed a molecular ion at *m/z* 188 along with a mass spectrum consistent with a substituted naphthoquinone (1,4-NQ + 30 amu), thus indicating a methoxy-NQ or a hydroxymethyl-NQ.

**Table 1:** Scent gland secretion compounds of *Metasiro savannahensis*: analytical data

Peak	RI	MS fragmentation (*m*/*z*)	Compound
D	1395	184, 169, 126, 97, 85, 71, 58(100), 43	n-dodecan-2-one
**E**	**1423**	**158(100), 130, 104, 102, 76**	**1,4-naphthoquinone**
F	1460	198, 183, 158, 71, 58(100), 43	Tridecan-2-one isomer (II)
G	1473	196, 178, 138, 125, 110, 96, 81, 68, 67, 54(100), 43	6-tridecen-2-one
H	1482	196, 178, 138, 125, 111, 97, 96, 81, 71(100), 67, 55, 43	7-tridecen-2-one
H1	1485	196*	Tridecenone-isomer
I	1487	194, 179, 151, 136, 125, 112, 107, 95, 93, 81, 79, 67(100), 55, 43	Tridecadienone
**J**	**1498**	**198, 183, 140, 71, 58(100), 43**	**n-tridecan-2-one**
J1?	1526	210*	Tetradecenone isomer
**L**	**1548**	**172(100), 157, 144, 118, 116, 115, 90, 89**	**6-methyl-1,4-naphthoquinone**
M	1561	212, 197, 152, 96, 71, 58(100), 43	Tetradecan-2-one isomer (II)
N	1568	212, 194, 172, 150, 79, 71, 58(100), 43	Tetradecan-2-one isomer (III)
O	1573	210, 152, 125, 124, 111, 110, 96, 95, 82, 81, 68(100), 54, 43	Tetradecenone isomer (II)
P	1583	?	?
Q	1598	212, 197, 169, 154, 96, 85, 71, 58(100), 43	n-tetradecan-2-one
R	1606	192/194*	4-chloro-1,2-naphthoquinone
**R1**	**1632**	**189(M + 1; 12), 188(100), 187(22), 173(1), 160(12), 134(6), 132(12), 131(15), 106(7), 105(4), 104(5), 103(4), 77(5)**	**5-hydroxy-6-methyl-1,4-naphthoquinone**
S	1666	222, 207, 179, 164, 140, 121, 111, 109, 107, 95, 93, 91, 80, 79(100), 67, 55, 43	Pentadecadienone isomer
T	1671	220, 202, 188, 173, 162, 133, 119, 108, 106, 105, 93, 79, 77, 43	Pentadecatrienone isomer
U	1675	224, 209, 206, 166, 138, 125, 124, 111, 110, 109, 97, 96, 95, 82, 81, 71, 69, 68, 67, 55, 54(100), 43	Pentadecenone isomer I
V	1680	224, 209, 206, 177, 166, 142, 125, 111, 96, 82, 81, 80, 79, 71, 69, 67, 58, 55, 54, 43(100)	Pentadecenone isomer II
V1	1688	224, 209, 206, 166, 142, 125, 111, 97, 96, 95, 93, 82, 81, 71(100), 69, 58, 55, 43	Pentadecenone isomer III
W	1699	226, 211, 208, 183, 168, 127, 96, 85, 71, 59, 58(100), 43	n-pentadecan-2-one
X	1736	206(100)/208, 193, 191, 180, 178. 171, 143, 115, 90, 89	4-chloro-6-methyl-1,4-naphthoquinone
Y	1761	238*	Hexadecenone-isomer

RI (retention index), according to Van den Dool and Kratz (1963). Compounds in bold are major constituents of the secretion (details in Table 2); compounds marked with * occurred in traces only, giving no clean mass spectral data

**Table 2:** A comparison of the composition of scent gland secretions in cyphophthalmids hitherto investigated

Peak	Compound	*Cyphophthalmus duricorius* (Sironidae)	*Siro exilis* (Sironidae)	Undetermined stylocellid (Stylocellidae)	*Austropurcellia forsteri* (Pettallidae)*	*Metasiro savannahensis* (Neogoveidae)
A	Acetophenone	0.45	–	–	–	–
B1	Undecenone (isomer)	–	–	–	0.15/0.23	–
**B**	**Undecan-2-one**	**9.71**	0.57	–	1.34/1.12	–
C	Dodecan-2-one (isomer)	1.66	0.34	–	0.43/0.34	–
D1	Dodecenone (isomer)	–	–	–	0.14/0.37	–
D	n-dodecan-2-one	2.01	0.89	–	3.58/5.80	0.6 ± 0.3
**E**	**1,4-naphthoquinone**	**17.61**	**14.01**	**2**	**20.84/19.33**	**18.6 ± 2.8**
F	Tridecan-2-one isomer (II)	1.00	0.59	–	0.46/0.51	0.3 ± 0.1
G	6-tridecen-2-one	4.02	4.13	–	3.18/3.41	3.2 ± 0.9
**H**	**7-tridecen-2-one**	**18.98**	**15.47**	–	**6.90/6.11**	**7.9 ± 2.6**
H1	Tridecenone-isomer	–	–	–	–	0.1 ± 0.2
I	Tridecadienone	3.27	0.65	–	3.70/6.26	0.3 ± 0.2
**J**	**n-tridecan-2-one**	**20.21**	**20.28**	**42**	**37.98/34.51**	**22.8 ± 2.8**
J1?	Tetradecenone isomer	–	–	–	–	0.1 ± 0.0
K	Tetradecan-2-one (isomer I)	0.06	0.17	–	0.16/0.09	–
**L**	**6-methyl-1,4-naphthoquinone**	**12.15**	**13.08**	1	**17.32/16.90**	**11.1 ± 1.3**
M	Tetradecan-2-one isomer (II)	0.52	1.15	8	1.29/1.67	0.9 ± 0.3
N	Tetradecan-2-one isomer (III)	0.65	0.45	–	0.25/0.24	0.3 ± 0.1
O	Tetradecenone isomer (II)	0.01	0.24	–	–	0.3 ± 0.1
P	Tetradecenone (?)	0.06	0.19	–	0.16/0.18	0.3 ± 0.1
Q	n-tetradecan-2-one	0.05	1.10	9	0.97/0.95	1.24 ± 0.3
**R**	**4-chloro-1,2-naphthoquinone**	**7.09**	**11.83**	–	0.46/0.97	0.1 ± 0.0
**R1**	**5-hydroxy-6-methyl-1,4-naphthoquinone**	–	–	–	–	**18.5 ± 1.8**
R2	6-methyl-1,4-naphthalenediol	–	–	1	–	–
S	Pentadecadienone isomer	0.04	3.00	8	–	3.4 ± 0.7
T	Pentadecatrienone isomer	0.05	0.92	–	–	0.8 ± 0.3
U	Pentadecenone (isomer I)	0.03	4.57	7	0.21/0.40	3.8 ± 0.9
V	Pentadecenone (isomer II ?)	0.01	0.37	3 (?)	–	1.2 ± 0.3
V1	Pentadecenone (isomer III)	–	–	3 (?)	–	0.5 ± 0.4
**W**	**n-pentadecan-2-one**	0.01	1.68	**14**	0.24/0.21	2.2 ± 0.8
X	4-chloro-6-methyl-1,4-naphthoquinone	0.36	4.30	–	0.24/0.92	1.4 ± 0.8
Y	Hexadecenone-isomer	–	–	–	–	0.1 ± 0.0
Z	2-methoxy-1,4-naphthoquinone	–	–	3	–	–

The table shows the relative abundance of compounds in the secretions of five cyphophthalmid species (calculated in % peak area of whole secretion). Data for *Cyphophthalmus duricorius* and *Siro exilis* are compiled from Raspotnig et al. (2005), for *Austropurcellia forsteri* from Raspotnig et al. (2012), and for the undetermined stylocellid from Jones et al. (2009). * For *A. forsteri*, data on male and female secretions (m/f) are given separately

For comparability, the original assignment of compounds by letters as introduced in Raspotnig et al. (2005) was maintained. In some cases, an accurate assignment (e.g., for compound P) was not possible; such compounds are marked with “?”. For *Metasiro*, data rely on five males and ten females; the detailed data for *C. duricorius, S. exilis* and *A. forsteri* (including standard deviations) can be found in the original publications. Main compounds (defined as of > 5% relative abundance) are in bold

Regarding methoxy-NQs, neither the mass spectrum nor the retention index of compound R1 (RI 1632) matched 2-methoxy-1,4-naphthoquinone (RI 1782), which is the only known methoxy-NQ isomer from the secretions of opilionids, namely from a stylocellid (Jones et al. 2009) and a few Dyspnoi (Raspotnig et al. 2010, 2014).

**Fig. 2:**
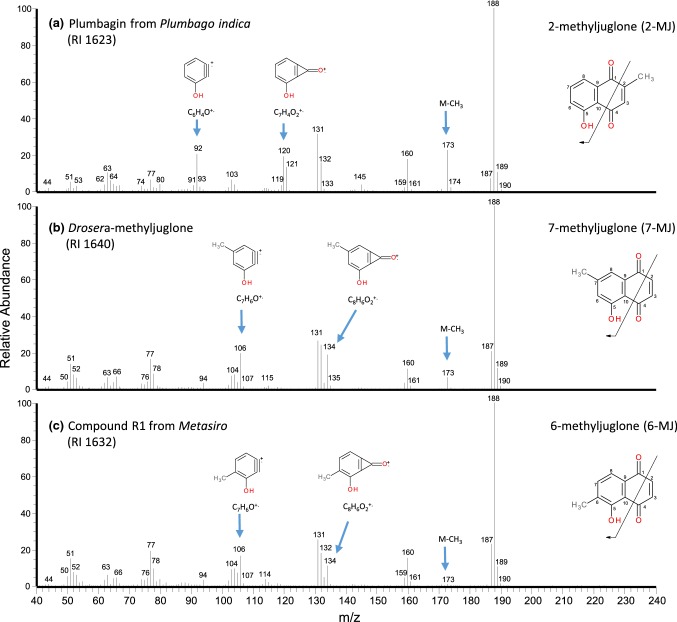
Mass spectra of naturally occurring methyljuglones and their distinguishing features: (**a**) plumbagin (2-methyljuglone) from *Plumbago indica*; (**b**) 7-methyljuglone from a *Drosera aliciae* cultivar; (**c**) 6-methyljuglone (compound R1) from *Metasiro savannahensis*

Regarding hydroxymethyl-NQs, particularly plumbagin and 7-methyljuglone (7-MJ) have already been identified from arthropod exocrine secretions (e.g., Sakata et al. 1997). A comparison of compound R1 to plumbagin showed clear differences in both retention time/index (RI 1623_plumbagin_ vs RI 1632_R1_) and mass spectrum (Fig. 2). According to Budzikiewicz et al. (1967), a major naphthoquinone fragmentation relies on the rupture of the C1–C2 and C4–C10 bonds. If the benzene ring carries the OH-group but not the methyl group, as in plumbagin (Fig. 2a), this rupture leads to a fragment ion at *m/z* 120, followed by the loss of CO. In this case, a fragment at *m/z* 92 arises (hydroxybenzyne-ion). On the other hand, compounds with both substituents (methyl- and hydroxy-group) on the benzene ring produce fragment ions at *m/z* 134 and *m/z* 106, respectively (Fig. 2b, c). These latter ions at *m/z* 134 (fragment from rupture of C1–C2 and C4–C10 bonds) and *m/z* 106 (hydroxymethyl-benzyne ion) have been reported for the spectrum of 7-MJ (e.g., Sakata et al. 1997) and were also observed in compound R1.

To compare compound R1 to authentic 7-MJ, a natural source for 7-MJ, namely an extract from leaves of a *Drosera* cultivar was used (Baranyai et al. 2016). The EI-mass spectrum of *Drosera*-derived 7-MJ indicated high, but not full correspondence with compound R1, mainly differing in the intensity of the fragment ion at *m/z* 173 (M-15: about 10% relative intensity in 7-MJ; less than 1% in compound R1) (Fig. 2b, c). Moreover, the retention time of 7-MJ was found to be slightly longer than for compound R1 (RI 1640_7-MJ_ vs RI 1632_R1_). If co-injected with a *Metasiro* extract, 7-MJ resulted in a peak eluting after compound R1, being clearly separated from it.

To check the potential identity of compound R1 with another isomer of methyljuglone, a GC–MS comparison of synthetic 6-methyljuglone (6-MJ) to compound R1 was carried out, finally proving full chromatographic and spectral correspondence (supplement 2). Compound R1 of the *Metasiro* extract was thus identified as 6-methyljuglone. The gas chromatographic separation of the three methyljuglones (plumbagin, 6-MJ, and 7-MJ) is shown in supplement 3.

### Sex specificity

Male and female secretion profiles showed no difference when compared by nMDS (supplement 4) and by PERMANOVA (pseudoF = 1.768; *n*_permutations_ = 9999; *p*_(same)_ = 0.1589). The profile of the single juvenile specimen exhibited the same blend of compounds as well. Due to the lesser quantity of the secretion in the juvenile, several compounds (basically all minor components in adults) were detectable in traces only.

### Direct sampling of secretion vs. whole body extracts

The whole bouquet of 25 components in the proportions described in Table 2 was consistently detected in whole body extracts of *M. savannahensis*. To test whether all of these components originated from the scent gland secretion of *M. savannahensis*, direct sampling of secretions on filter paper immediately after discharge from ozopores was carried out. A comparison to whole body extracts revealed no qualitative differences. However, amounts of dabbed secretions were generally lower, and minor compounds of whole body extracts were retrieved in filter extracts in traces only, making filter paper extracts—at least statistically—distinguishable from whole body extracts (pseudoF = 12.33; *n*_permutations_ = 9999; *p*_(same)_ = 0.0001) (supplement 4). From experiments with *Cyphophthalmus duricorius*, we know that the scent gland secretion is never extruded entirely, but in small portions, making many discharge events possible. It is indeed very difficult to experimentally empty glands completely by repeated mechanical disturbance; small amounts of secretion are always retained. Whole body extraction, however, leads to a full wash-out of secretion directly into the solvent, and therefore, is the method of choice when collecting secretion from cyphophthalmids. Interestingly, body surface components such as long chain hydrocarbons—as present in many other arthropods—were never found in these extracts.

### Biological role of 6-MJ and occurrence in animals and plants

Regarding the biological significance of opilionid secretions, a general defensive function has been postulated (see introduction), and indeed experimentally been proven for a few species (Eisner et al. 2004). With respect to leg dabbing against offenders (Juberthie 1961), chemical defense by cyphophthalmid scent glands is obvious; interestingly, in species of *Cyphophthalmus*, the secretion also spreads all over the body, hence impregnating the body surface, a phenomenon that has been observed in *Metasiro* as well. Naphthoquinones have been ascribed antimicrobial properties for many decades (e.g., Thomson 1971), and thus it is likely that the secretion of *Metasiro* additionally provides broad protection against bacteria and fungi. This latter function may be of uppermost importance when inhabiting a microorganism-rich habitat such as the deep layers of leaf litter.

Generally, naphthoquinones are not as widespread as other defensive compounds in arthropods: apart from opilionids, only some beetles and some millipedes are known to produce naphthoquinonic components (Blum 1981; Dettner 1993; Shear 2015). Hydroxy-naphthoquinones in animals are particularly scarce. Compounds such as juglone (5-hydroxy-1,4-naphthoquinone), plumbagin, and 7-MJ are well known from different plants, mainly from *Juglans*, *Plumbago*, *Euclea*, *Diospyros* and *Drosera* (e.g., Babula et al. 2009), where these are known to exert allelopathic effects. In animals, there are reports for juglone, plumbagin and 7-MJ from the defensive secretions of some chrysomelid larvae (Matsuda and Sugawara 1980), phlaeothripine Thysanoptera (Suzuki et al. 1995, 2004) and from an uropodine mite (Sakata et al. 1997). To the best of our knowledge, 6-MJ has not been reported from any animal source yet. From the plant *Euclea natalensis*, 6-MJ has been described in the context of HIV-1 reverse transcriptase inhibition (Tshikalange et al. 2007).

For juglone, plumbagin and 7-MJ, a number of antibiotic properties have already been reported, including antigermination, antibacterial, antifungal and general cytotoxic activities (Khan et al. 1978; Spencer et al. 1986; Didry et al. 1994; Rahalison et al. 1994; Inbaraj and Chignell 2004). Regarding 6-MJ in the secretion of *Metasiro* in particular, consistent defensive and antibiotic roles are supposed. Initial studies with the synthetic compound proved antibacterial activity against *Mycobacterium tuberculosis* (Mahapatra et al. 2007) and cytotoxicity against several cancer cell lines (Kishore et al. 2014).

### Chemosystematic considerations

*Metasiro savannahensis*is the fifth species of Cyphophthalmi and the first of the family Neogoveide that has chemically been investigated. In total, 33 components have so far been identified from cyphophthalmid secretions. The secretion of *Metasiro* resembles the chemistry known from Sironidae, Stylocellidae and Pettalidae with respect to its general organization into naphthoquinones and methyl ketones. However, the naphthoquinone content in *Metasiro* is relatively high, clearly dominating the secretion. In contrast to Sironidae, but like in Pettalidae and Stylocellidae, chloro-naphthoquinones do not contribute much to this naphthoquinone-fraction (Raspotnig et al. 2005, 2012; Jones et al. 2009). A comparison of secretions from all five species hitherto analyzed is shown in Table 2.

Cyphophthalmids are considered the sister group to the remaining Opiliones (i.e., the suborders Eupnoi, Dyspnoi, and Laniatores). Interestingly, many cyphophthalmid compounds can be retrieved in the exudates of other harvestmen suborders, and thus such compounds are perhaps symplesiomorphies of all harvestmen, possibly reflecting an ancestral character state of opilionid secretions. This may be the case with some naphthoquinones, such as 1,4-naphthoquinone and 6-methyl-1,4-naphthoquinone, which are also found in phalangiid Eupnoi and nemastomatid Dyspnoi (e.g., Wiemer et al. 1978; Raspotnig et al. 2010, 2014). Even the unusual chloro-naphthoquinones—originally considered an autapomorphy of cyphophthalmid secretions (Raspotnig et al. 2005)—have since been detected in certain species of Dyspnoi (e.g., Raspotnig et al. 2014). Only 6-MJ, as here identified from *M. savannahensis*is, has not yet been found in any other opilionid. Whether it represents an autapomorphy of *Metasiro*, or whether it characterizes further neogoveids or even other cyphophthalmids, remains unknown. Methyl ketones, on the other hand, may represent an autapomorphic character set in cyphophthalmid secretions. These compounds have been reported from all cyphophthalmid species so far analyzed, including *Metasiro*, but are not common in the other opilionid suborders. Schaider et al. (2018) recently hypothesized a common ancestry for methyl ketones of cyphophthalmids and acyclic compounds (mainly ethyl ketones) of many Eupnoi and Dyspnoi. This concept is intriguing, and indeed some evidence has since emerged (e.g., an intermediate methyl-/ethyl ketone chemistry in some dyspnoans) that supports this idea (Raspotnig et al. 2014; Schaider et al. 2018).

For cyphophthalmid chemosystematics and chemotaxonomy, the discovery of 6-MJ in *Metasiro* adds to the growing list of naphthoquinone derivatives in mite harvestmen. These compounds may help to support relationships of cyphophthalmid families. According to Giribet et al. (2012), Pettalidae is sister to all other Cyphophthalmi, with Neogoveidae (together with Ogoveidae and Troglosironidae) as sister to Sironidae + Stylocellidae. Fernandez et al. (2017) also found an early divergence of Pettallidae, but then Sironidae + Neogoveidae as sister to Stylocellidae. In either of these scenarios, both the well-known opilionid naphthoquinones (1,4-naphthoquinone and 6-methyl-1,4-naphthoquinone) and the chloro-naphthoquinones appear to be ancestral in cyphophthalmids. 1,4-Naphthoquinone and 6-methyl-1,4-naphthoquinone represent major compounds in all species analyzed, whereas chloro-naphthoquinones—according to a single study by Jones et al. (2009)—may be lacking in stylocellids. This lack, however, may be interpreted as a regression. 6-MJ, in an evolutionary context, certainly represents a derivative cyphophthalmid naphthoquinone. If found in other neogoveids and possibly other families, 6-MJ may be considered a valuable synapomorphic character in a clade of derived cyphophthalmid taxa.

## Electronic supplementary material

Below is the link to the electronic supplementary material.

Supplementary material 1 (PDF 205 kb)

Supplementary material 2 (PDF 177 kb)

Supplementary material 3 (PDF 134 kb)

Supplementary material 4 (PDF 199 kb)
