# Supplementary material for: The scent gland chemistry of neogoveid cyphophthalmids (Opiliones): an unusual methyljuglone from Metasiro savannahensis
Source: Chemoecology. 2019 Sep 26;29(5):189–97. doi: 10.1007/s00049-019-00288-y (PMC6884433; doi:10.1007/s00049-019-00288-y)
Supplement: Supplementary file 1 — Supplementary material 1 (PDF 205 kb) [file 49_2019_288_MOESM1_ESM.pdf]

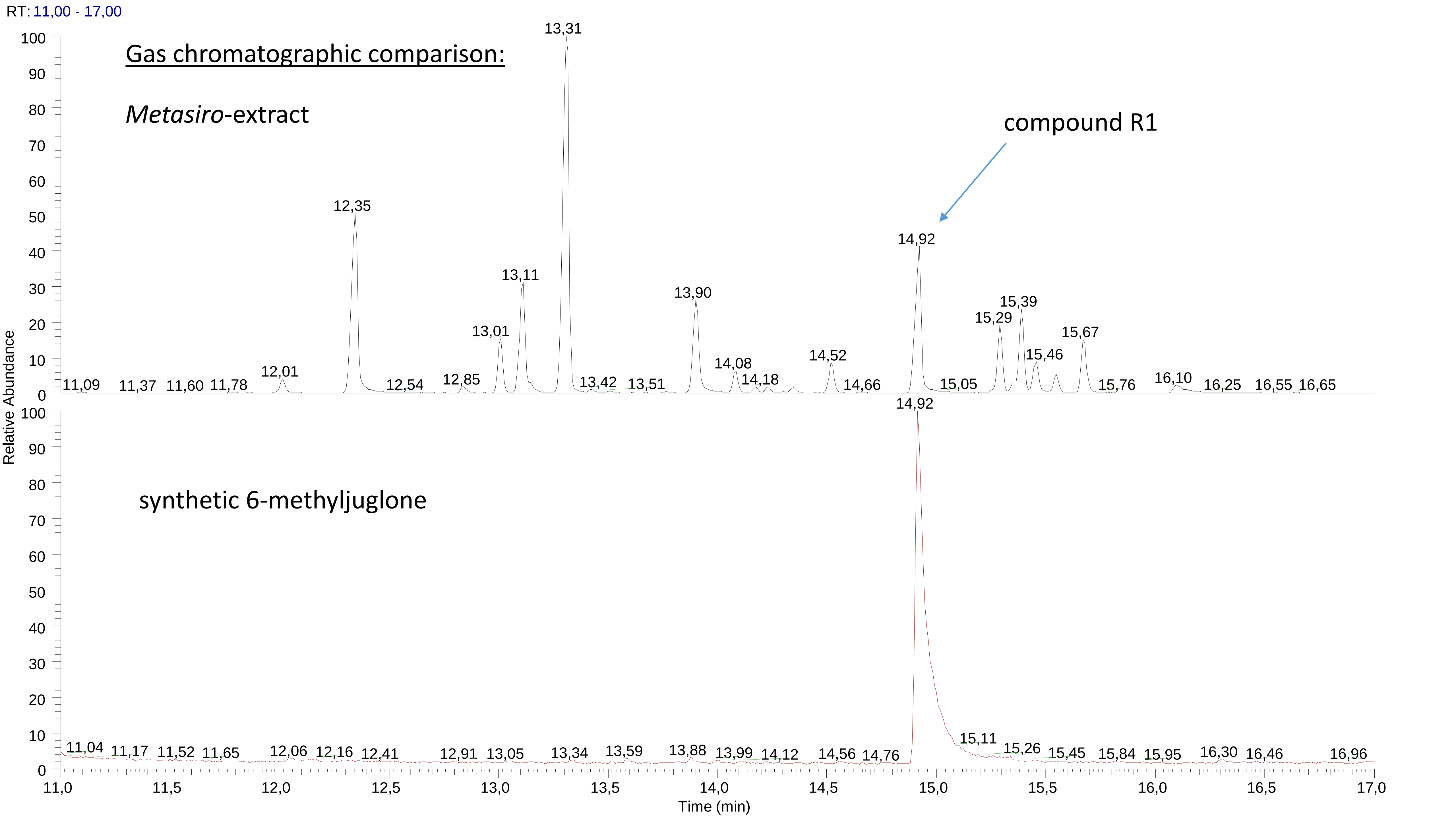

RT: 11,00 - 17,00
Gas chromatographic comparison:
Metasiro-extract
compound R1
synthetic 6-methyljuglone
Relative Abundance
Time (min)
13,31
12,35
14,92
13,11
13,90
15,39
15,29
15,67
13,01
14,52
15,46
14,08
12,01
14,18
16,10
12,85
13,42
13,51
14,66
15,05
15,76
16,25
16,55
16,65
11,09
11,37
11,60
11,78
12,54
15,11
15,26
15,45
11,04
11,17
11,52
11,65
12,06
12,16
12,41
12,91
13,05
13,34
13,59
13,88
13,99
14,12
14,56
14,76
15,84
15,95
16,30
16,46
16,96

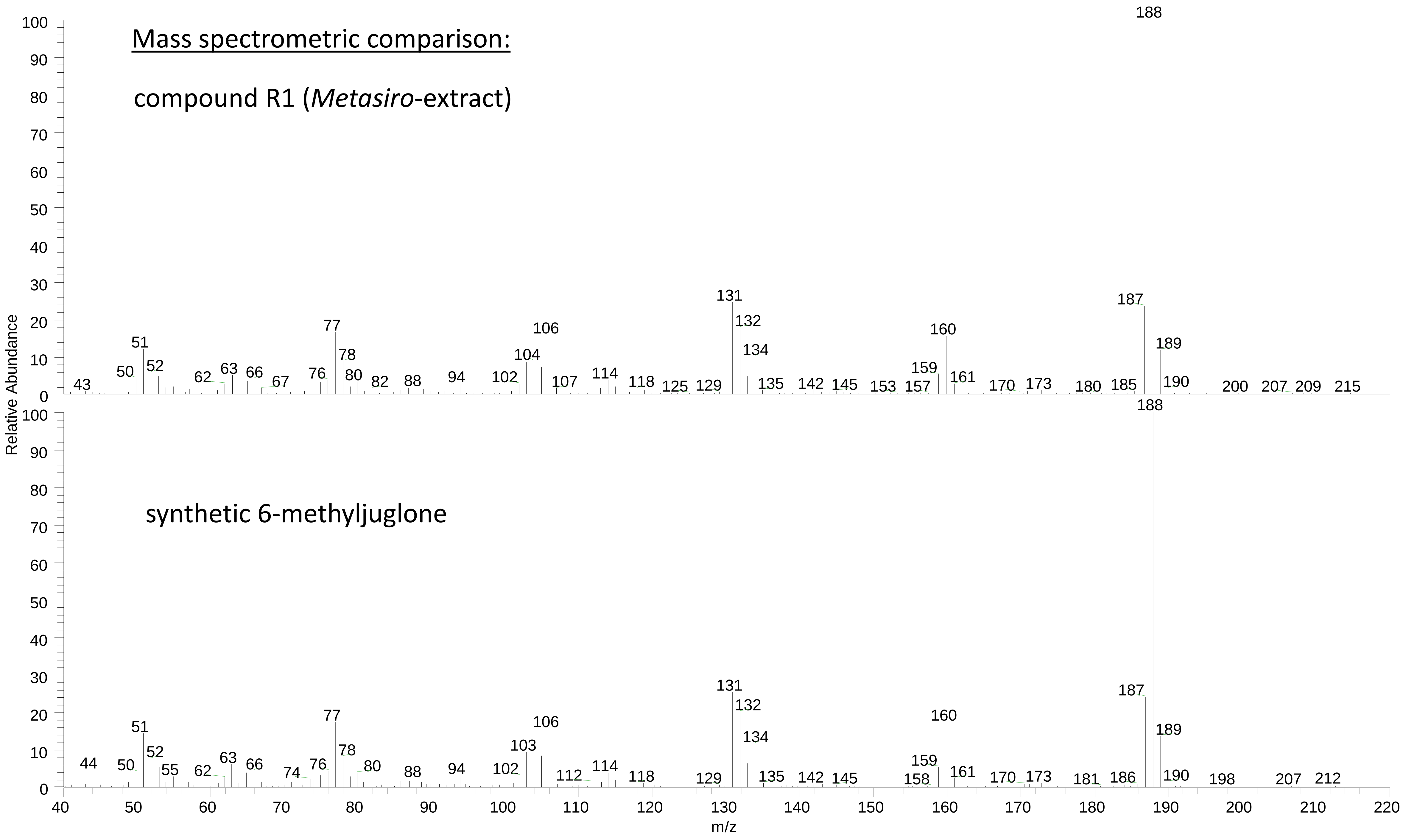
Mass spectrometric comparison:
compound R1 (*Metasiro*-extract)
synthetic 6-methyljuglone
Relative Abundance
m/z
188
187
131
132
134
160
159
189
106
104
77
78
51
52
50
43
62
63
66
67
76
80
82
88
94
102
107
114
118
125
129
135
142
145
153
157
161
170
173
180
185
190
200
207
209
215
103
44
55
74
112
158
181
186
198
212
