# Supplementary figures and images for: The scent gland chemistry of neogoveid cyphophthalmids (Opiliones): an unusual methyljuglone from Metasiro savannahensis
Source: Chemoecology. 2019 Sep 26;29(5):189–97. doi: 10.1007/s00049-019-00288-y (PMC6884433; doi:10.1007/s00049-019-00288-y)

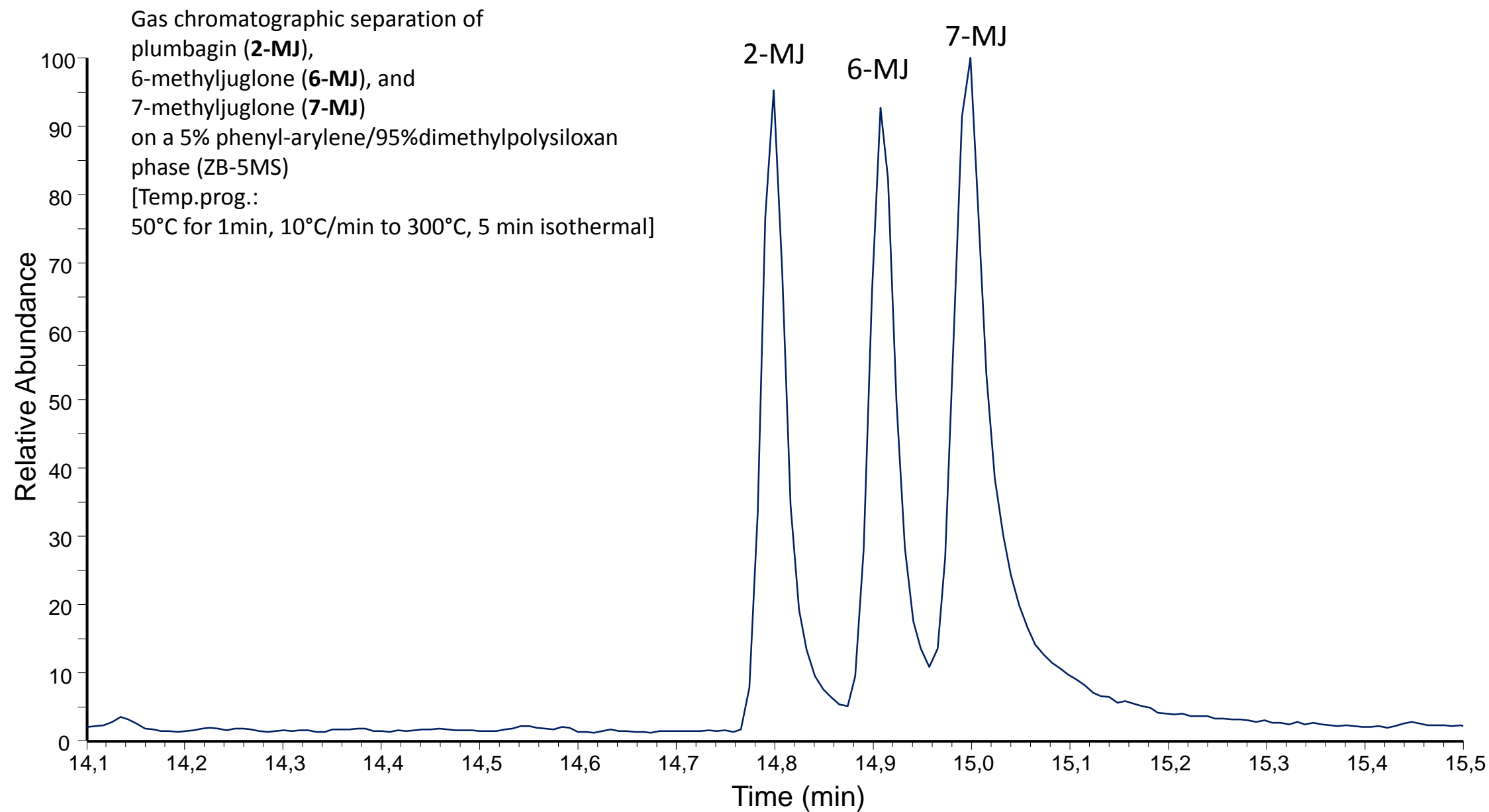

Supplement: Supplementary file 2 — Supplementary material 2 (PDF 177 kb) [file 49_2019_288_MOESM2_ESM.pdf]
