# Supplementary material for: The scent gland chemistry of neogoveid cyphophthalmids (Opiliones): an unusual methyljuglone from Metasiro savannahensis
Source: Chemoecology. 2019 Sep 26;29(5):189–97. doi: 10.1007/s00049-019-00288-y (PMC6884433; doi:10.1007/s00049-019-00288-y)
Supplement: Supplementary file 3 — Supplementary material 3 (PDF 134 kb) [file 49_2019_288_MOESM3_ESM.pdf]

Supplement 4:

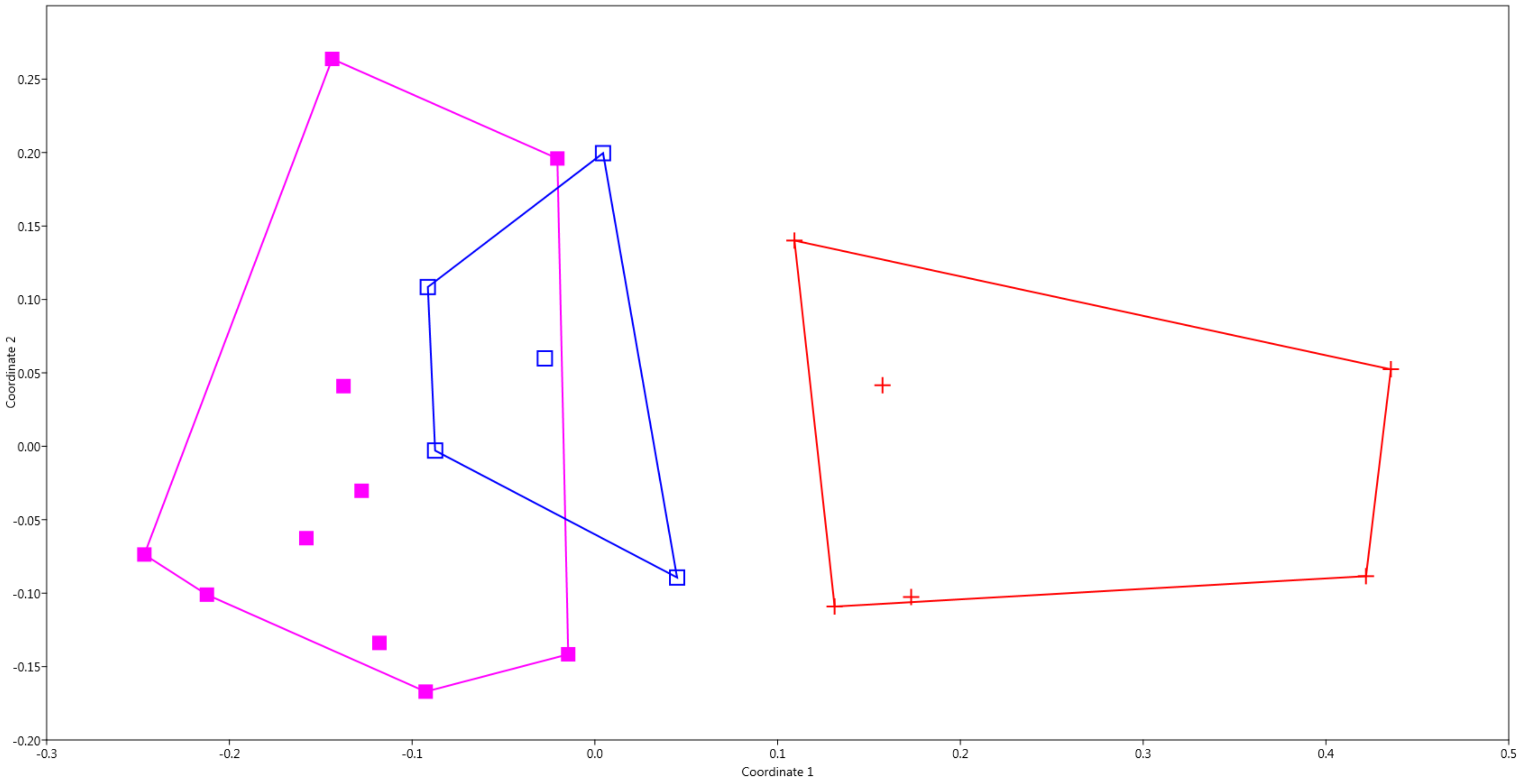

nMDS plot (Bray-Curtis dissimilarity) calculated from individual adult profiles shows no clear separation between the profiles of whole body extracts of different sexes, but separation from profiles obtained by filter-paper dabbing (males: blue squares; females: pink filled squares; filter paper: red crosses).
