# Supplementary material for: The scent gland chemistry of neogoveid cyphophthalmids (Opiliones): an unusual methyljuglone from Metasiro savannahensis
Source: Chemoecology. 2019 Sep 26;29(5):189–97. doi: 10.1007/s00049-019-00288-y (PMC6884433; doi:10.1007/s00049-019-00288-y)
Supplement: Supplementary file 4 — Supplementary material 4 (PDF 199 kb) [file 49_2019_288_MOESM4_ESM.pdf]

Supplement 1:

Proton (700MHz) and carbon (175 MHz) NMR shift values of plumbagin, 6-MJ and 7-MJ in $CDCl_3$ at 25 °C, TMS as internal standard. *J* in Hz.

atom	plumbagin		6-MJ		7-MJ	
	δ_C	δ_H	δ_C	δ_H	δ_C	δ_H
1	184.8	-	184.3	-	184.6	-
2	149.6	-	136.9*	6.92 (s)	138.8	6.92 (s)
3	135.5	6.80 (q, 1.6)	138.6*	6.92 (s)	139.3	6.91 (s)
4	193.3	-	190.7	-	189.8	-
4a	115.1	-	114.2	-	113.0	-
5	161.2	-	160.1	-	161.7	-
6	124.2	7.25 (dd, 8.2, 1.3)	135.3	-	124.2	7.09 (brs)
7	136.1	7.60 (t, 7.6)	136.0	7.49 (d, 7.7)	148.5	-
8	119.3	7.63 (dd, 7.5, 1.3)	119.0	7.54 (d, 7.7)	120.5	7.44 (brs)
8a	132.1	-	129.7	-	131.5	-
2-Me	16.5	2.19 (brs)				
6-Me			16.1	2.35 (s)		
7-Me					22.3	2.44 (s)
5-OH	-	11.98 (s)	-	12.24 (s)	-	11.83 (s)

The resonances marked with an "*" are exchangeable.
